# Temporary Tumor Perfusion Changes From Subcutaneous Lidocaine With 1% Epinephrine During Particle Embolization for Meningiomas

**DOI:** 10.7759/cureus.82624

**Published:** 2025-04-20

**Authors:** Chihara Hideo, Taichi Ikedo, Masahiro Sawada, Takayuki Kikuchi, Yoshiki Arakawa

**Affiliations:** 1 Department of Neurosurgery, Kyoto University, Kyoto, JPN

**Keywords:** embosphere, epinephrine adverse effects, meningioma embolization, particle embolization, lidocaine

## Abstract

Preoperative embolization of meningiomas aims to minimize intraoperative bleeding and improve surgical outcomes. Effective embolization requires an embolic material to reach the tumor’s feeding arteries. In particle embolization, particles travel via arterial blood flow to occlude feeding vessels within the tumor. To enhance embolization, we temporarily reduced transosseous blood flow from the cutaneous artery by inducing contraction of the superficial temporal artery (STA) and occipital artery (OA) through subcutaneous injection of lidocaine with epinephrine. A patient with a meningioma involving the superior sagittal sinus underwent preoperative embolization. A microcatheter was inserted into the middle meningeal artery (MMA), and angiography revealed tumor staining with late-phase backflow from perfusion pressure in other feeders. Using roadmap images from external carotid angiography, clustered cutaneous artery areas were identified. Subcutaneous injection of 1% lidocaine with epinephrine was administered, resulting in expanded tumor staining and restored antegrade blood flow via the MMA. Embolization using Embosphere particles followed. Postoperative angiography showed reduced tumor enhancement, with no side effects from the injection. Subcutaneous lidocaine with epinephrine effectively suppresses the dominant transosseous blood supply, enhancing tumor embolization. This technique appears promising for improving preoperative outcomes.

## Introduction

Preoperative embolization of meningiomas is widely regarded as a safe and effective adjunct to surgery. However, its effects on postoperative outcomes have been limited [1,2]. Studies have reported reduced surgical bleeding, decreased need for blood transfusions, and shorter operative times when adequate intra-tumoral embolization was achieved [3,4]. Furthermore, embolization has been associated with improved long-term outcomes, including recurrence-free survival [3-5].

High convexity, parasagittal, and superior sagittal sinus meningiomas often have transosseus feeders from the superficial temporal artery (STA) or occipital artery (OA) [6]. In these cases, embolization through the middle meningeal artery (MMA) can be complicated by blood flow from transosseous feeders, preventing embolic material from reaching the tumor and leading to incomplete embolization.

To address this, various methods have been reported to improve the embolic effect by controlling the blood flow from transosseous feeders, when considering embolization from MMA [7-10]. In this report, we present a case of subcutaneous injection of lidocaine with epinephrine to temporarily reduce blood flow from transosseous feeders, enhancing the intratumoral embolic effect during particle embolization with Embosphere via the MMA.

## Case presentation

**Figure 1:**
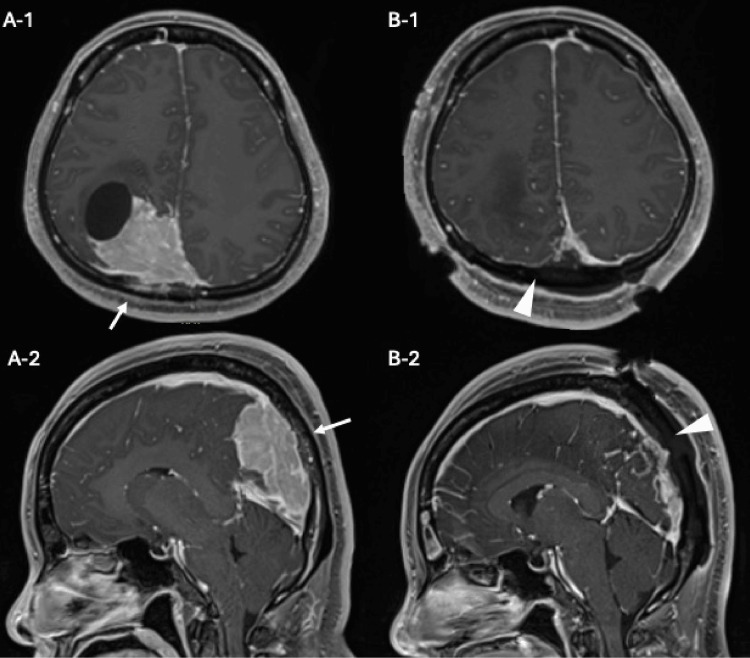
Contrast-enhanced MRI findings (A-1) Axial and (A-2) sagittal contrast-enhanced MRI images reveal a cystic tumor located near the sigmoid sinus in the parietal region, demonstrating uniform enhancement (white arrow). The tumor is observed invading the sigmoid sinus. (B-1) Axial and (B-2) sagittal contrast-enhanced MRI images post-craniotomy show successful removal of the tumor, with no visible brain damage (white arrow head).

A 47-year-old female presented with a superior sagittal sinus meningioma, identified during an evaluation for persistent headaches (Figure 1).

**Figure 2:**
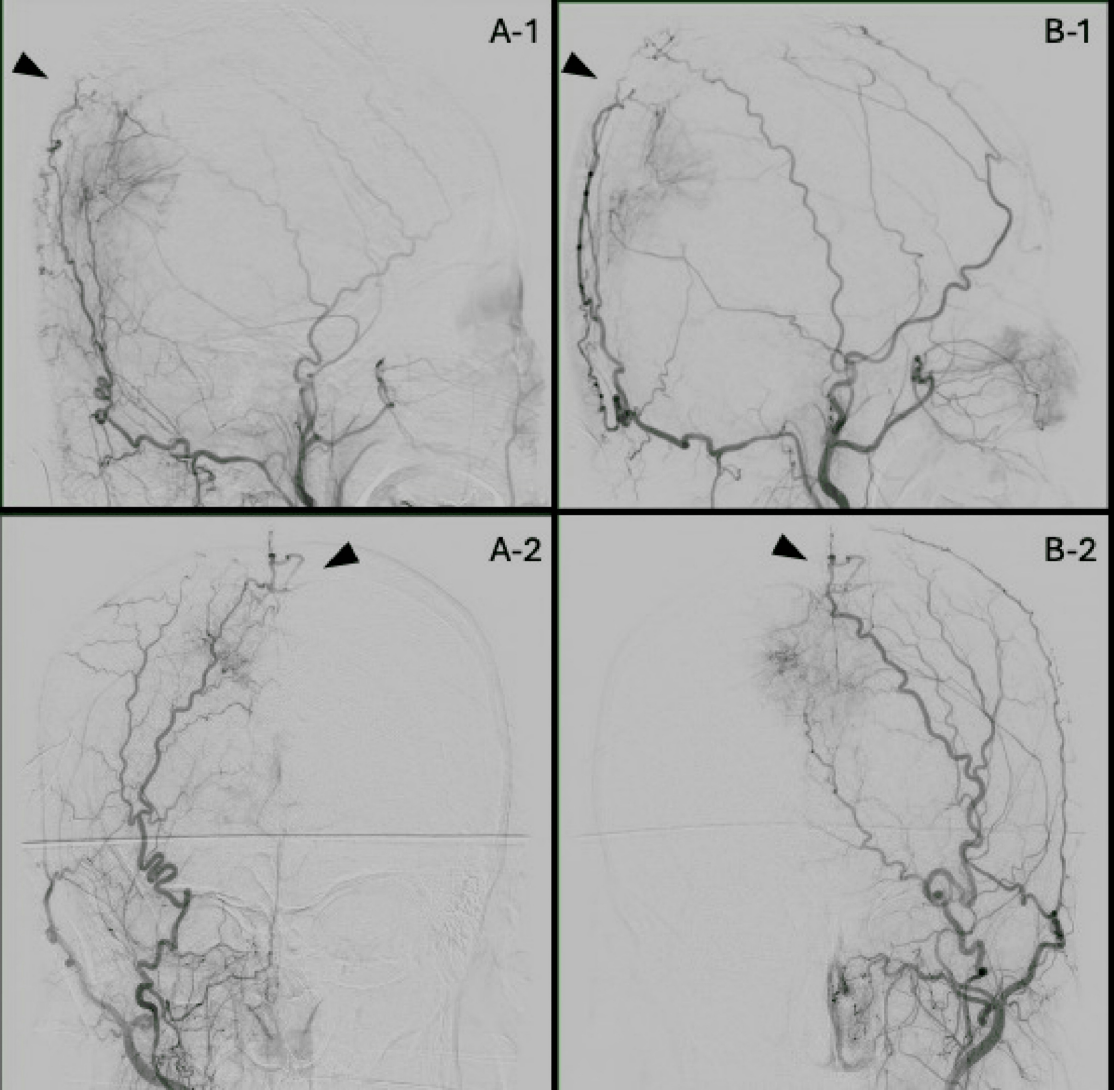
Findings of the external carotid artery angiography (A-1) Lateral and (A-2) PA views of the right external carotid artery angiography, along with (B-1) lateral and (B-2) PA views of the left external carotid artery angiography, demonstrate tumor feeding from the bilateral middle meningeal arteries (MMA). In addition, abundant transosseous feeders (black arrow head) from the bilateral superficial temporal arteries (STA) and bilateral occipital arteries (OA) are confirmed.

Cerebral angiography revealed significant transosseous feeders from the bilateral OA and STA, in addition to bilateral MMA and the right tentorial artery (Figure 2). 

Super-selective angiography using an SL-10 microcatheter (OD: 0.022 inch, ID: 0.0165 inch, Stryker, Kalamazoo, MI, USA) guided into the right MMA demonstrated reflux of contrast medium into the accessed MMA during the late phase (Video 1). To address this, we identified the transosseous entry points of the STA and OA feeders using biplanar external carotid artery angiography (ECAG) with roadmap guidance. Subcutaneous injection of 20 mL of 1% lidocaine with epinephrine (1:100,000) was administered around the sites where the feeders were observed penetrating the skull in the calvarial region. The injection sites were carefully targeted based on anatomical correlation from orthogonal roadmap views to ensure accurate delivery. The injection was performed gradually, and ECAG was repeated as necessary to confirm a reduction in tumor blush from the transosseous feeders.

**Video 1:** Reflux of the contrast medium into the accessed middle meningeal artery (MMA) during the late phase

**Figure 3:**
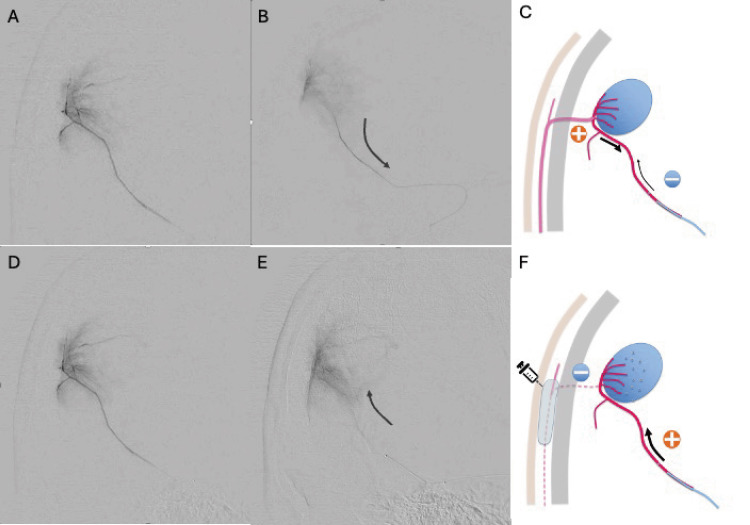
Selective angiography findings before and after subcutaneous injection of epinephrine-added lidocaine (A) Early phase of selective angiography after guiding the microcatheter into the posterior convexity branch of the middle meningeal artery (MMA), showing tumor stain. (B) In the delay phase, reflux into the accessed feeder is observed. (C) This phenomenon is thought to result from reduced blood flow in the MMA branch due to the microcatheter, leading to a relative increase in transosseous blood flow from the occipital artery (OA) and other areas. Subsequently, transosseous blood flow was reduced by subcutaneous injection of epinephrine-added lidocaine, causing a relative increase in blood flow in the MMA branch, shifting to antegrade blood flow (F). In the post-treatment selective angiography of the MMA, blood flow towards the tumor is observed in both the early (D) and delay (E) phases, with no reflux into the MMA feeder.

Following the injection, right ECAG showed reduced blood flow in the OA and STA and relatively increased blood flow in the MMA feeder (Video 2). Subsequent super-selective angiography from the same site confirmed contrast enhancement within the tumor and the absence of backflow (Figure 3).

**Video 2:** Right external carotid artery angiography (ECAG) showing reduced blood flow in the occipital arteries (OA) and superficial temporal arteries (STA) and relatively increased blood flow in the middle meningeal artery (MMA) feeder

**Figure 4:**
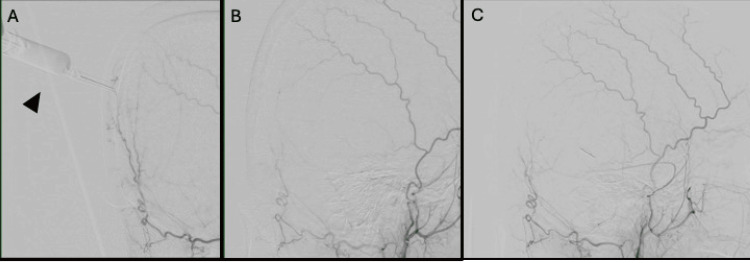
Right external carotid artery angiography findings (A) Right external carotid artery angiography identifies the site where the occipital artery (OA) and superficial temporal artery (STA) converge as trans-osseous feeders. A subcutaneous injection of lidocaine with epinephrine (black arrow head) is administered at this site. (B) Post- subcutaneous injection of lidocaine with epinephrine angiography shows decreased definition in the periphery of the STA and OA, confirming reduced blood flow. (C) After embolization, external carotid artery angiography (ECAG) confirms the disappearance of tumor stain.

Embolization was then performed using Embosphere particles (100-300 μm) (Figure 4).

**Figure 5:**
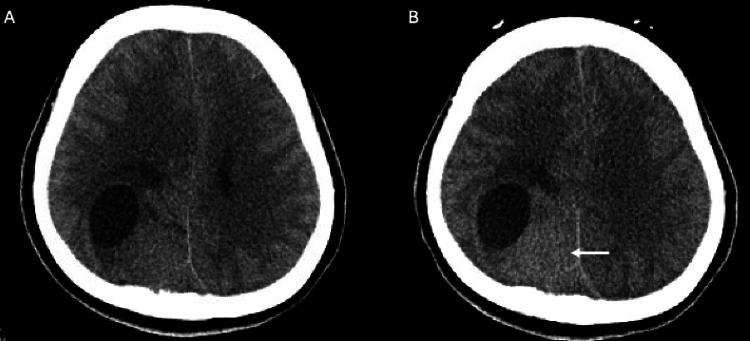
CT findings (A) Non-contrast CT image obtained prior to embolization, demonstrating a tumor with cystic components. (B) CT image obtained four hours after embolization. Although four hours had elapsed, the contrast agent entrapped within the tumor remained stagnant without washout (white arrow).

A delayed CT scan, taken four hours post-procedure, showed pooling of contrast medium, confirming extensive embolization within the tumor and reduced intra-tumoral blood flow (Figure 5).

Two days later, the patient underwent tumor resection and was discharged on postoperative day 12 without any deficits. The extent of resection was Simpson grade II, and no tumor recurrence has been observed during the one-year postoperative follow-up period (Figure 1).

Approval for this study was obtained from the Institutional Review Board of Kyoto University. The original approval documents are in Japanese, and the IRB reference number is R2088. The study was conducted in accordance with the principles of the Declaration of Helsinki.

## Discussion

In this case, the blood flow of the transosseous feeder from the cutaneous artery was temporarily reduced by subcutaneous injection of lidocaine containing epinephrine, which enhanced the effect of tumor embolization by Embosphere from MMA and made it possible to safely remove the tumor.

While preoperative embolization does not directly improve Simpson grade or overall surgical outcomes [1], it may offer other benefits, such as prolonging the recurrence-free interval [2] without increasing complications [5].

Adequate intra-tumoral embolization is recommended, as studies have reported reduced intraoperative bleeding and lower blood transfusion requirements when complete embolization is achieved. In order to improve the efficacy of intra-tumoral embolization, there have been reports on the selection of embolization materials and target feeders, as well as on methods to increase tumor perfusion from the feeders for embolization.

Embolization material comparison

In comparisons between NBCA and Embosphere, no significant differences in resection time, intraoperative blood loss, or Simpson grade have been observed. The difference lies primarily in the surgeon's perception of "dryness" or "softness" during surgery, with Embosphere often preferred because it is less likely to affect the elasticity of the tumor [11]. The injection methods differ between particulate embolic materials and liquid embolic materials. Unlike particulate embolic agents such as Embosphere, which are highly dependent on antegrade blood flow to reach and occlude the target vasculature, liquid embolic materials like NBCA or Onyx can be delivered in a more controlled fashion [7]. These agents can penetrate the tumor vasculature even against the direction of flow, depending on the injection technique, and are therefore less influenced by hemodynamic conditions. For this reason, effective blood flow control is extremely important when using Embosphere for tumor embolization.

Feeder selection for embolization

Parasagittal and convexity meningiomas are more likely to achieve complete embolization [3,11]. However, meningiomas fed by the transosseous can hinder effective embolization from the MMA due to competing blood flow from transosseous feeders. Selective embolization of transosseous feeders is often challenging due to the tortuosity of the vessels, and extensive occlusion can reduce skin blood flow, leading to ischemic necrosis and delayed wound healing [12]. Therefore, the MMA feeder is typically selected for embolization in most meningioma cases.

Techniques for flow control of transosseous feeders

When transosseous feeders are present, particles may not reach the tumor through the injection of Embosphere from MMA, resulting in inadequate embolization. 

Various techniques have been reported to control transosseous feeder flow. Manual STA compression [10], the use of bilateral external carotid artery flow control during transarterial embolization [8], and subcutaneous injection of lidocaine with epinephrine [9] (as in this case) have all been reported as effective in a dural arteriovenous fistula case. The effect of transseus feeder blood flow suppression is not permanent for each technique. This is to avoid necrosis due to poor blood flow in the skin and delayed wound healing after surgery.

Balloon-guiding catheter (BGC)-assisted embolization has been reported as another strategy for temporary flow control, particularly in cases with significant transosseous supply [8]. While this technique enables real-time, adjustable occlusion of the external carotid artery (ECA), it simultaneously reduces flow through the middle meningeal artery (MMA), potentially compromising the forward delivery of particulate embolic agents. In contrast, our approach selectively reduces flow from transosseous feeders without diminishing MMA perfusion, thereby enhancing the effectiveness of MMA-directed embolization. Additionally, our method offers a simpler setup with fewer systemic effects and lower procedural cost.

In the present case, we used 1% lidocaine with epinephrine, a solution commonly utilized for local anesthesia. Using this technique, we injected the epinephrine-containing lidocaine around the transosseous feeders, previously identified using external carotid artery angiography, to reduce blood flow via vasoconstriction. A relatively large volume of 20 mL was administered, which caused the skin to become taut, further decreasing blood flow through mechanical compression. The lidocaine component helped alleviate the discomfort associated with the tightness by providing local analgesia, thereby reducing the patient’s overall distress. The transient effects of the injection ensure that normal blood flow to the skin resumes after embolization, minimizing the risks of skin necrosis and delayed wound healing. This approach balances effective blood flow modulation with patient safety and comfort.

Limitations

This is a case report, and the described technique may not be universally applicable. The lack of post-embolization contrast-enhanced MRI prevented quantitative assessment of embolization extent. In addition, the outcomes were not compared with those of simple middle meningeal artery (MMA) embolization, so the additional benefit of the technique remains unproven. Furthermore, as this method restores tumor perfusion via transosseous feeders following embolization, there is a potential risk of intratumoral bleeding. Further studies with larger cohorts are required to evaluate the incidence of post-embolization tumor bleeding.

Other potential complications also warrant consideration. Although no adverse events were observed in this case, subcutaneous injection of lidocaine with epinephrine carries theoretical risks, including skin necrosis, delayed wound healing, and systemic effects such as hypertension, tachycardia, or arrhythmia. While the vasoconstrictive effect of epinephrine is temporary and unlikely to impact surgical resection performed hours or days later, systemic safety cannot be fully established based on a single case. Additional investigation is needed to assess the safety profile of this technique in broader clinical use.

## Conclusions

The subcutaneous injection of 1% lidocaine with epinephrine may help reduce transosseous arterial supply from cutaneous branches in meningioma cases with significant transosseous feeders. This technique appears to increase the relative perfusion territory of the middle meningeal artery, potentially enhancing the effectiveness of particle embolization via the MMA. Based on this case, the method is technically straightforward and was performed without observed complications; however, further studies are needed to assess its safety and efficacy more robustly.
